# The Dynamic Motor Control Index as a Marker of Age-Related Neuromuscular Impairment

**DOI:** 10.3389/fnagi.2021.678525

**Published:** 2021-07-22

**Authors:** Ashley N. Collimore, Ashlyn J. Aiello, Ryan T. Pohlig, Louis N. Awad

**Affiliations:** ^1^Neuromotor Recovery Laboratory, Department of Physical Therapy, College of Health and Rehabilitation Sciences: Sargent College, Boston University, Boston, MA, United States; ^2^Biostatistics Core Facility, University of Delaware, Newark, DE, United States

**Keywords:** aging, gait, muscle activity, motor control, walking

## Abstract

Biomarkers that can identify age-related decline in walking function have potential to promote healthier aging by triggering timely interventions that can mitigate or reverse impairments. Recent evidence suggests that changes in neuromuscular control precede changes in walking function; however, it is unclear which measures are best suited for identifying age-related changes. In this study, non-negative matrix factorization of electromyography data collected during treadmill walking was used to calculate two measures of the complexity of muscle co-activations during walking for 36 adults: (1) the number of muscle synergies and (2) the dynamic motor control index. Study participants were grouped into young (18–35 years old), young-old (65–74 years old), and old–old (75+ years old) subsets. We found that the dynamic motor control index [χ^2^(2) = 9.41, *p* = 0.009], and not the number of muscle synergies [χ^2^(2) = 5.42, *p* = 0.067], differentiates between age groups [χ^2^(4) = 10.62, *p* = 0.031, Nagelkerke *R*^2^ = 0.297]. Moreover, an impairment threshold set at a dynamic motor control index of 90 (i.e., one standard deviation below the young adults) was able to differentiate between age groups [χ^2^(2) = 9.351, *p* = 0.009]. The dynamic motor control index identifies age-related differences in neuromuscular complexity not measured by the number of muscle synergies and may have clinical utility as a marker of neuromotor impairment.

## Introduction

During adult aging, physiological changes lead to impaired muscle strength (Janssen et al., 2002; Doherty, 2003; Lauretani et al., 2003; Goodpaster et al., 2006; King et al., 2016; Morrison and Newell, 2019), increased muscular atrophy (Janssen et al., 2002; Doherty, 2003; Lauretani et al., 2003; Goodpaster et al., 2006), and reduced neuromuscular control (i.e., impaired muscle recruitment and coordination) (Clark et al., 2010; Dingwell et al., 2017; Morrison and Newell, 2019; Kara et al., 2020). As a result, older adults tend to have poorer balance (King et al., 2016; Cruz-Jimenez, 2017), walk at slower speeds (Menz et al., 2003; Kim and Kim, 2014; Cruz-Jimenez, 2017), and take fewer steps per day (Bassett et al., 2010) than younger adults. These deficits continue to progress with further aging; endurance (Gardener et al., 2006; Schrack et al., 2016), walking speed (Xie et al., 2017; Morrison and Newell, 2019), balance (Xie et al., 2017), and muscle mass (Doherty, 2003; Castell et al., 2013; Rong et al., 2020) significantly decrease with increasing age from 50 to 80+ years old. Untreated, these impairments can lead to significantly reduced participation in the community (Allison et al., 2013; Warren et al., 2016) and an increased risk of falling (Verghese et al., 2009; Toebes et al., 2012; Ambrose et al., 2013; Lusardi et al., 2017), one of the leading causes of morbidity among older adults (Burns and Kakara, 2018).

For older adults, the evaluation and treatment of mobility impairments are centered on clinical data collected from functional assessments, such as gait speed and the short performance physical battery (Middleton and Fritz, 2013; Soubra et al., 2019). These assessments quantify *observed* functional deficits and can track recovery with rehabilitation (The LIFE Study Investigators, 2006; Brach and Vanswearingen, 2013); however, prognostic biomarkers that can *predict* age-related functional decline have the potential to promote healthier aging by triggering early, targeted interventions that can mitigate or reverse the mobility deficits that contribute to a higher fall risk and reduced quality of life. Recent evidence suggests that among older adults, measurable changes in neuromuscular control precede functional changes (Clark et al., 2013; Dingwell et al., 2017). Thus, the incorporation of neuromuscular control measurements into routine clinical evaluations may enable timely identification of emerging deficits.

Common measures of neuromuscular control evaluate the relationship between the descending inputs from the central nervous system and coordination of muscular outputs (Ivanenko et al., 2004; Clark et al., 2013; Kamp et al., 2013; Palmer et al., 2016; Liu et al., 2019; Rozand et al., 2019; Awad et al., 2020; Opie et al., 2020). A popular measure of neuromuscular control that quantifies the coordinated co-activation of muscles during walking is a muscle synergy analysis, with different muscle synergy metrics, such as the number of synergies and the composition and timing of those synergies, used to express different aspects of control in young adults (Ivanenko et al., 2004; Chvatal and Ting, 2013), older adults (Sawers and Bhatt, 2018; Baggen et al., 2020; Santuz et al., 2020), and individuals with neurological diagnoses (Clark et al., 2010; Ting et al., 2015). The number of muscle synergies that underlie a motor task has consistently been used as a measure of the complexity of neuromuscular control and is associated with functional abilities (Furuya and Altenmüller, 2013; Sawers et al., 2015; Yaserifar et al., 2021) and fall risk (Allen and Franz, 2018; Sawers and Bhatt, 2018). These findings are often used to motivate using the number of synergies to characterize deficits in the neuromuscular control of walking.

Although the number of muscle synergies differentiates between the extremes of skill and impairment, a wide range of clinical presentations are found among individuals with the same number of muscle synergies. Indeed, older adults do not show a reduction in the number of muscle synergies compared to younger adults (Monaco et al., 2010; Baggen et al., 2020; Santuz et al., 2020), despite known walking deficits (Menz et al., 2003; Kim and Kim, 2014; Cruz-Jimenez, 2017). This suggests that the number of muscle synergies may not be suitable for the detection of age-related impairments in the neuromuscular control of walking. More recently, the dynamic motor control index—a summary metric of muscle co-activations during walking—has emerged as an alternative measure of neuromuscular complexity (Steele et al., 2015; Schwartz et al., 2016; Shuman et al., 2019). This measure computes the variability accounted for (VAF) from the one-synergy solution and scales it to a z-score based on a reference group (see section “Materials and Methods”), with a higher index representing higher neuromuscular complexity (Steele et al., 2015). By expressing neuromuscular complexity in this way, the dynamic motor control index is an intuitive measure that can be quickly interpreted by both researchers and clinicians. Previous work has shown that the dynamic motor control index can differentiate between neurologically intact children and children with cerebral palsy assessed at different levels of the Gross Motor Functional Classification System and Gillette Functional Assessment Questionnaire (Steele et al., 2015). Recently, da Silva Costa et al. (2020) demonstrated that the one-synergy VAF differs between younger and older adults performing complex walking balance tasks, whereas the number of muscle synergies does not.

To build upon this work, the primary aim of this study was to determine if, compared to the number of muscle synergies, the dynamic motor control index (i.e., a scaled version of the one-synergy VAF) could better quantify age-related differences in neuromuscular control during treadmill walking. We examined the index as both a continuous predictor of age-related changes and a categorical predictor of impairment based on a cutoff of one standard deviation lower than controls. We hypothesized that the dynamic motor control index would be better than the number of muscle synergies at differentiating between younger and older adults, as well as between young-old and old–old subgroups. We also hypothesized that as a categorical predictor of impairment, the dynamic motor control index would identify differences in the number of impaired individuals across increasing age-groups.

## Materials and Methods

### Participants

Data from 38 adults were gathered from a publicly available data set (Santuz et al., 2020). Inclusion criteria included age between 18 and 80 years old and no known neurological impairments. Participants were grouped by age into younger (<35 years old; YA) and older (≥65 years old; OA) adults. The older adults were then subdivided into two groups: young-old (<75 years old; YO) and old–old (≥75 years old; OO). These subsets have been shown to differ in amount of sedentary time (Kim and Lee, 2019), quality of life (Kim and Lee, 2019), and prevalence of frailty and chronic conditions (Fried et al., 2001; Hayutin and Dietz, 2010). All procedures were approved by the Ethics Committees of the Humboldt-Universität zu Berlin, Kassel University, and Heidelberg University.

### Data Collection

**FIGURE 1:**
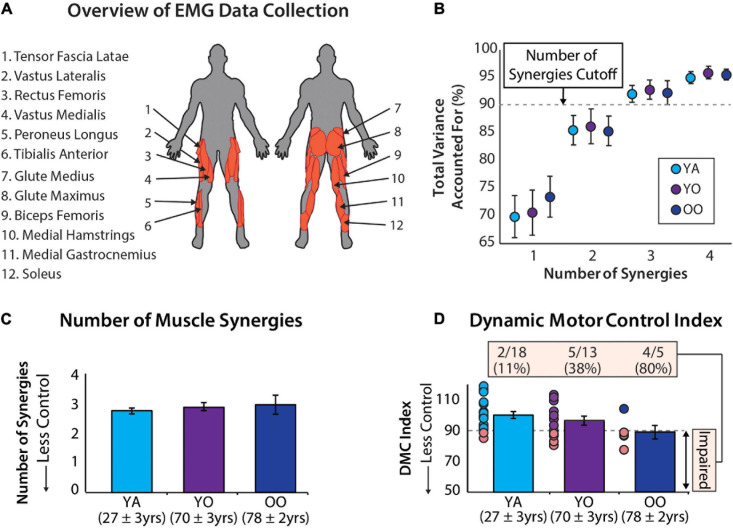
**(A)** Muscle activity was measured unilaterally from younger and older adults from 12 muscles. **(B)** Average ± standard deviation of the total variance accounted for by one to four synergies for each group. **(C)** The number of muscle synergies does not differentiate between age groups. Errors bars are standard error. Mean age ± standard deviation are shown for each group. **(D)** The dynamic motor control index does differentiate between age groups and identifies a significantly different percentage of individuals with impaired neuromuscular complexity between groups [χ^2^(2) = 9.35, *p* = 0.009]. Errors bars are standard error. Mean age ± standard deviation shown for each group.

Data collection procedures are detailed by Santuz et al. (2020). For our analyses, we extracted raw EMG data from 1 min of treadmill walking (i.e., the second unperturbed walking trial from experimental protocol E3) and re-analyzed these data for all participants. The younger cohort walked at 1.2 m/s and the older cohort walked at 1.1 m/s, based on the average comfortable walking speed for younger and older adults from a pilot study (Santuz et al., 2020). Electromyography data were collected from 12 right lower limb muscles (Figure 1A): vastus medialis, rectus femoris, vastus lateralis, tensor fascia latae, soleus, medial gastrocnemius, peroneus longus, tibialis anterior, biceps femoris, medial hamstrings, gluteus maximus, and gluteus medius.

### Data Processing

EMG signals for all participants were processed using custom MATLAB scripts. The data were high-pass filtered at 40 Hz using a 4th order Butterworth filter, de-meaned, rectified, low-pass filtered at 4 Hz using a 4th order Butterworth filter, and resampled to 1000 Hz. Strides containing signal artifacts were removed from the EMG data through visual inspection, and the last 30 clean strides were used for subsequent analyses. Each stride was then resampled to 101 points to normalize to percentage of the gait cycle. Following visual inspection, the tensor fascia latae was removed for all participants due to poor data quality.

The number of muscle synergies was calculated for each participant using non-negative matrix factorization with a modified version of a MATLAB script made publicly available by Ting and Chvatal (2010). The cutoff criteria for the number of muscle synergies was set to 90% of the variability in data accounted for (VAF) or until the addition of a new synergy did not increase the total VAF by more than 5%. Dynamic motor control index values were also calculated using non-negative matrix factorization, but the total number of allowable synergies was constrained to one. Constraining the number of muscle synergies to one minimizes the impact of pre-processing and cutoff criteria, which have been shown to be variable across research groups and produce different results in the number of muscle synergies (Ivanenko et al., 2004; Coscia et al., 2015; Shuman et al., 2017; Kieliba et al., 2018). As a result, the dynamic motor control index has high potential to serve as a robust summary metric of muscle co-activation during walking.

The one-synergy VAF values were converted into dynamic motor control indices using the following equation first presented by Steele et al. (2015), with young-adults serving as the control group:

$$D\,y\,n\,a\,m\,i\,c\,M\,o\,t\,o\,r\,C\,o\,n\,t\,r\,o\,l\,I\,n\,d\,e\,x=100+10\,\left(\frac{A\,V\,G\,V\,A\,F_{1\,s\,y\,n\,e\,r\,g\,y-C\,o\,n\,t\,r\,o\,l}-V\,A\,F_{1\,s\,y\,n\,e\,r\,g\,y-E\,x\,p}}{S\,D\,V\,A\,F_{1\,s\,y\,n\,e\,r\,g\,y-C\,o\,n\,t\,r\,o\,l}}\right)$$

where AVG VAF_1synergy–Control_ is the average of the VAF of the one-synergy solution of the young adult control group, SD VAF_1synergy–Control_ is the standard deviation of the VAF of the one-synergy solution of the young adult control group, and VAF_1synergy–Exp_ is the VAF of the one-synergy solution for each individual in the experimental groups (i.e., young-old and old–old adults).

### Data Analysis

Binomial logistic regression was used to test if the dynamic motor control index was significantly predictive of age group (young or old) after adjusting for the number of muscle synergies. The first block contained the main effects of muscle synergies and the dynamic motor control index, and the second block included their interaction. The same predictors were used in a multinomial logistic regression comparing younger, young-old, and old–old groups. The old–old group was used as the reference comparison in the multinomial logistic regression. The number of muscle synergies and dynamic motor control index were both mean-centered.

A chi-square test examined the differences in the number of individuals classified as impaired across the three age groups. An individual was considered impaired if they had a dynamic motor control index value more than one-standard deviation less than the mean of the young adult group (i.e., <90).

## Results

Data for two individuals were removed from all analyses; the first was a young adult who had high signal artifacts across several muscles, and the second was an older adult who was identified as an outlier based on examination of residuals, leaving 36 study participants. The primary analyses were conducted with and without the single outlier, without a notable effect on the results. On average, the young adults (*N* = 18; 11 female) were 27 ± 3 years old and the older adults (*N* = 18; 13 female) were 72 ± 5 years old. After subdividing the older group into those younger than and older than 75 years old, the young-old subgroup (*N* = 13; 10 female) was 70 ± 3 years old and the old–old subgroup (*N* = 5; 3 female) was 78 ± 2 years old, on average.

The total variance accounted for by 1 to 4 synergies is reported for the young, young-old, and old-old groups in Figure 1B. Using the cutoff criteria described in *Data Processing*, these groups had, on average, 2.78 ± 0.43, 2.92 ± 0.49, and 3.0 ± 0.71 muscle synergies (Figure 1C) and a dynamic motor control index of 100 ± 10, 96.4 ± 10.79, and 88.9 ± 9.67 (Figure 1D), respectively. In total there were 7 individuals with two muscle synergies, 27 individuals with three muscle synergies, and 2 individuals with four muscle synergies. Additionally, 12 participants had a dynamic motor control index below our impairment threshold of 90. Individual subject results are reported in Table 1.

**TABLE 1:** Participant characteristics and neuromuscular complexity.

**Participant**	**Subgroup**	**Sex**	**Age**	**# of muscle synergies**	**Dynamic motor control index**
H1	YA	F	22	3	98.320
H2	YA	F	24	3	92.847
H3	YA	F	24	3	102.397
H4	YA	F	25	3	91.030
H5	YA	F	25	3	91.202
H6	YA	F	25	3	99.074
H7	YA	F	25	3	99.549
H8	YA	F	25	3	107.443
H9	YA	F	26	2	84.930
H10	YA	F	26	3	93.793
H11	YA	M	27	2	91.863
H12	YA	M	28	3	116.856
H13	YA	M	29	3	119.134
H14	YA	M	30	3	88.414
H15	YA	M	30	2	101.512
H16	YA	F	31	2	97.930
H17	YA	M	33	3	115.174
H18	YA	M	35	3	108.533
H19	YO	F	65	2	89.996
H20	YO	F	65	3	80.554
H21	YO	F	68	3	97.565
H22	YO	F	69	3	102.564
H23	YO	F	69	3	113.427
H24	YO	F	70	3	82.939
H25	YO	F	70	3	97.489
H26	YO	F	71	2	86.748
H27	YO	F	71	3	93.341
H28	YO	M	72	3	87.949
H29	YO	M	72	3	99.794
H30	YO	M	72	4	109.551
H31	YO	F	74	3	111.707
H32	OO	M	76	3	86.600
H33	OO	F	77	2	77.533
H34	OO	F	78	3	87.195
H35	OO	M	80	3	88.712
H36	OO	F	80	4	104.281

The logistic regression model predicting dichotomous age group by the main effects of muscle synergies and the dynamic motor control index was significant [χ^2^(2) = 6.20, *p* = 0.045, Nagelkerke *R*^2^ = 0.211]. The dynamic motor control index significantly predicted age group (*p* = 0.039, OR = 1.089), while the number of synergies was not significant (*p* = 0.077, OR = 0.222). When added to the model, the interaction between the two variables was also not significant (*p* = 0.239). Model results are presented in Table 2.

**TABLE 2:** Binomial logistic regression results: younger and older adults.

**Model statistics**					**Predictor statistics**			
	**Model**	***R*^2^**	***X*^2^**	***p***	**Predictors**	**β**	**OR**	***p***
Model 1	Muscle synergies and dynamic motor control index	0.211	6.20	0.045	Constant	0.013	1.013	0.971
					Muscle synergies	–1.507	0.222	0.077
					Dynamic motor control index	0.085	1.089	0.039
Model 2	Muscle synergies, dynamic motor control index, and interaction	0.303	9.283	0.026	Constant	0.366	1.442	0.409
					Muscle synergies	–2.602	0.074	0.144
					Dynamic motor control index	0.100	1.105	0.029
					Muscle synergies × dynamic motor control index	–0.218	0.804	0.239

The multinomial logistic regression model predicting subgroups of young, young-old, and old–old by the main effects of muscle synergies and dynamic motor control index, was significant [χ^2^(4) = 10.62, *p* = 0.031, Nagelkerke *R*^2^ = 0.297]. Again, the dynamic motor control index was significant [χ^2^(2) = 9.41, *p* = 0.009] while the number of synergies was not [χ^2^(2) = 5.42, *p* = 0.067]. Specifically, after adjusting for the number of synergies, the dynamic motor control index was significantly positively predictive of being in the old–old age group compared to the young age group (Wald χ^2^ = 5.16, *p* = 0.023, OR = 1.26), but was not significant for the young-old group (Wald χ^2^ = 2.97, *p* = 0.085, OR = 1.19). When added to the model, the interaction between the number of synergies and the dynamic motor control index was not significant (*p* = 0.060). Model results are presented in Table 3.

**TABLE 3:** Multinomial logistic regression results: young, young-old, and old–old adults.

**Model statistics**					**Subgroup analysis (OO reference group)**					
	**Model**	***R*^2^**	***X*^2^**	***p***		**Subgroup**	**Predictors**	**β**	**OR**	***p***
Model 1	Muscle synergies and dynamic	0.297	10.62	0.031	Model 1	YA	Constant	2.297		0.023
	motor control index						Muscle synergies	–3.369	0.034	0.068
Model 2	Muscle synergies, dynamic motor	0.422	16.251	0.012			Dynamic motor control index	0.230	1.258	0.023
	control index, and interaction					YO	Constant	2.045		0.044
**Predictor statistics**							Muscle synergies	–2.194	0.111	0.227
							Dynamic motor control index	0.169	1.185	0.085

	**Predictors**	**X ^2^**	***p***							

Model 1	Constant	11.266	0.004		Model 2	YA	Constant	3.863		0.020
	Muscle synergies	5.416	0.067				Muscle synergies	–5.835	0.003	0.034
	Dynamic motor control index	9.412	0.009				Dynamic motor control index	0.342	1.408	0.020
Model 2	Constant	16.309	0.000				Muscle synergies × dynamic motor	–0.357	0.700	0.098
	Muscle synergies	9.452	0.009				control index			
	Dynamic motor control index	12.561	0.002			YO	Constant	3.336		0.043
	Muscle synergies × dynamic motor	5.631	0.060				Muscle synergies	–3.678	0.025	0.092
	control index						Dynamic motor control index	0.268	1.307	0.060
							Muscle synergies × dynamic motor control index	–0.156	0.855	0.182

Using a dynamic motor control index impairment threshold of 90 to identify individuals with impairment in neuromuscular complexity, we observed between-group differences in the number of impaired individuals [χ^2^(2) = 9.351, *p* = 0.009]. More specifically, 11.1% of young adults, 38.5% of young-old adults, and 80% of old–old adults were impaired (Figure 1D).

## Discussion

The primary finding of this study is that the dynamic motor control index captures changes in neuromuscular control with progressing age that the number of independent muscle synergies does not. Consistent with our hypothesis, the number of muscle synergies was not a significant indicator of age group, even when sub-dividing older adults into young-old and old–old groups. In partial support of our hypothesis, the dynamic motor control index was able to differentiate between younger adults and all older adults, and differentiate between the old–old and young adult subgroups, but was not able to differentiate between young-old and old–old subgroups.

Our findings build on previous work which showed that the one-synergy VAF (i.e., unscaled dynamic motor control index) of electromyography data collected during complex walking balance tasks is significantly different between younger and older adults (da Silva Costa et al., 2020). The high task difficulty of the balance tasks (i.e., walking on a narrow piece of tape and walking on a narrow beam) is thought to have magnified differences in neuromuscular control across the age groups. In contrast, our results suggest that even a simple treadmill walking task can identify impairments in neuromuscular control in older adults, particularly those older than 75 years. However, our failure to observe a significant difference between the young-old and old–old subgroups may be due to the simplicity of the treadmill walking task. The timely identification of emerging age-related deficits may require evaluating neuromuscular control across different walking tasks that range in complexity.

The proportion of individuals classified as having a neuromuscular impairment (i.e., those with a dynamic motor control index less than 90) was significantly different across age groups (Figure 1D). These results not only demonstrate the ability of the dynamic motor control index to identify increasing neuromuscular impairment across age groups, but also highlight the interpretability and potential clinical utility of this metric. By scaling each person’s one-synergy VAF to a z-score, clinicians can quickly identify a meaningful neuromuscular impairment based on the difference from 100 (i.e., the average score of the reference group). Classification based on the one-synergy VAF is also possible but would be much less intuitive. Our study suggests that a cutoff of 90 may be useful for screening for age-related neuromuscular impairment. Interestingly, two individuals in the young adult group were classified as “impaired” based on their dynamic motor control indices being less than 90. Without corroborating clinical data, it is not known if these two young adults have actual neuromuscular impairments or if this finding is a limitation in the specificity of the metric. Indeed, because our threshold for impairment (i.e., a score of 90) is one standard deviation from the mean of the young adult group, by definition some young adults will be classified as “impaired.” Substantial future work is needed to evaluate the diagnostic accuracy of different dynamic motor control index impairment thresholds. Anchoring the impairment threshold on statistical, clinical, or patient-perceived criteria and using these to derive small, moderate, and large meaningful difference scores would further improve clinical usability.

The number of muscle synergies has been used as a measure of motor ability (Furuya and Altenmüller, 2013; Sawers et al., 2015; Yaserifar et al., 2021) and impairment (Allen and Franz, 2018; Sawers and Bhatt, 2018); however, its suitability for the detection of age-related neuromuscular impairment is questionable (Monaco et al., 2010; Baggen et al., 2020; da Silva Costa et al., 2020; Santuz et al., 2020). Thus, a primary goal for this study was to evaluate if the dynamic motor control index could better quantify age-related differences in neuromuscular complexity. As a continuous summary metric of muscle activity during walking, the dynamic motor control index highly contrasts with the discrete nature of the number of muscle synergies. That is, unlike the dynamic motor control index, the number of muscle synergies identified during walking spans a relatively small range of values, resulting in low measurement resolution and a limited ability to distinguish between individuals. This weakness has required examination of other muscle synergy metrics [e.g., analysis of the weightings and timings of the synergies (Steele et al., 2017; Jacobs et al., 2018; Baggen et al., 2020; Santuz et al., 2020)] to identify neuromuscular impairments among those with the same number of synergies, but even those metrics are limited in their ability to identify age-related neuromuscular impairments (Monaco et al., 2010).

An additional benefit of the dynamic motor control index is that it is not influenced by filtering approach and cutoff criteria in the same ways as the number of muscle synergies (Shuman et al., 2017; Kieliba et al., 2018). In the literature, the number of synergies reported during walking ranges from 3 to 7, with 4 synergies as the most common finding (Ivanenko et al., 2004; Chvatal and Ting, 2013; Sawers et al., 2015; Allen and Franz, 2018). These differences are heavily influenced by the number and type of muscles studied, filtering techniques, and cutoff criteria (Ivanenko et al., 2004; Coscia et al., 2015; Shuman et al., 2017; Kieliba et al., 2018). Our finding of three synergies for the majority of the adults included in our study is similar to the results of Steele et al. (2017) and Coscia et al. (2015). Moreover, Monaco et al. (2010) reported that three primary synergies accounted for most of the variance in muscle activity during walking, with additional synergies identified by the algorithm accounting for only an additional 15% of the variance without providing additional information about muscle coordination. While the average number of synergies observed in our study sample is on the lower end of the range reported in the literature, our results are consistent with prior research showing no significant difference in the number of synergies between older and younger adults (Monaco et al., 2010; Baggen et al., 2020; da Silva Costa et al., 2020; Santuz et al., 2020). Methodological differences across studies highlight the need for more standardized approaches for muscle synergy analyses. To that end, whereas the number of muscle synergies is often not comparable across research groups, the dynamic motor control index (which can be derived from the same data used to compute the number of synergies) has potential to serve as a more universal approach to measuring neuromuscular complexity.

Although limited in the ability to distinguish among individuals with the same number of synergies, prior work has shown that a reduced number of synergies can identify individuals at the extremes in neuromuscular complexity, including older adults with a history of falls (Allen and Franz, 2018; Sawers and Bhatt, 2018). Indeed, individuals in our study with only two independent muscle synergies were more likely to have an impaired dynamic motor control index (i.e., four of seven, 57%) compared to individuals with three muscle synergies (i.e., seven of 27, 26%). Moreover, although not a significant independent predictor in either of our regression models, the number of synergies approached significance in both the binomial (*p* = 0.077) and multinomial (*p* = 0.067) models. These results suggest that the number of muscle synergies and the dynamic motor control index may offer synergistic information about neuromuscular complexity and future work is needed to further investigate this relationship.

### Limitations

There are several limitations of this study. Although previous research has shown high variability in the number of synergies observed during walking across older adults (e.g., Clark et al., 2010), it is noteworthy that 75% of the participants in our study had three synergies. Re-analysis in a more heterogeneous cohort may be valuable. However, it should be noted that although our study sample may be homogenous in terms of the number of muscle synergies, it is *not* homogenous in terms of the age-related neuromuscular impairment that is captured by the dynamic motor control index.

While the results of this study demonstrate the ability of the dynamic motor control index to identify changes in neuromuscular control with age, studying its usefulness as a measure to identify walking impairments prior to a functional decline is beyond the scope of this study and will be investigated in future work. Finally, we focused on the number of synergies, which is used as a measure of neuromuscular complexity, and not the muscle composition or timing of the individual synergies. These may be useful in characterizing neuromuscular control deficits in a way that the dynamic motor control index cannot. Ultimately, a combination of neuromuscular control metrics may be necessary to fully characterize impairments.

## Conclusion

The dynamic motor control index can identify age-related differences in the complexity of muscle activations during walking that the number of muscle synergies does not. Moreover, a dynamic motor control index of 90 appears to be a meaningful threshold indicator of age-related neuromotor impairment. Together, these findings support evaluation of the dynamic motor control index’s potential to facilitate timely detection of age-related functional decline. Additionally, the findings of this study show that lower extremity electromyography data from a simple treadmill walking task are sufficient to differentiate between age groups, a finding of importance to advancing the overall accessibility of approaches to measuring neuromuscular control deficits.

## Data Availability Statement

Electromyography data were made publicly available by Santuz et al. (2020). The original contributions presented in the study are included in the article/supplementary material, further inquiries can be directed to the corresponding author/s.

## Ethics Statement

This study involving human participants was reviewed and approved by Humboldt-Universität zu Berlin, Kassel University, and Heidelberg University. The patients/participants provided their written informed consent to participate in this study.

## Author Contributions

AC and LA designed the study and research questions. AC wrote the code and analyzed the data. AA reviewed all the code. AC and RP completed the statistical analyses. AC, AA, RP, and LA interpreted the results and drafted the manuscript. All authors contributed to the article and approved the submitted version.

## Conflict of Interest

The authors declare that the research was conducted in the absence of any commercial or financial relationships that could be construed as a potential conflict of interest.
